# Relationship of Para and Perirenal Fat and High-Density Lipoprotein and Its Function in Patients with Type 2 Diabetes Mellitus

**DOI:** 10.1155/2021/9286492

**Published:** 2021-12-22

**Authors:** Jing Ke, Yan Wang, Simo Liu, Kun Li, YueChao Xu, Longyan Yang, Dong Zhao

**Affiliations:** ^1^Center for Endocrine Metabolism and Immune Diseases, Beijing Luhe Hospital, Capital Medical University, Beijing 101149, China; ^2^Beijing Key Laboratory of Diabetes Research and Care, Beijing 101149, China

## Abstract

**Background:**

Para and perirenal fat is a fat pad surrounding the kidneys. Recent studies showed the association between para and perirenal fat and cardiovascular diseases including atherosclerosis and hypertension. We aimed to assess the relationship between para-perirenal ultrasonographic fat thickness and serum high-density lipoprotein (HDL) level and cholesterol efflux capacity of HDL in patients with type 2 diabetes mellitus (T2DM).

**Methods:**

We recruited 58 subjects with T2DM and collected anthropometric indices including height, weight, waist circumference, and other clinical data. Para-perirenal ultrasonographic fat thickness (PUFT) was measured via ultrasound. Serum lipid profile and other metabolic indices were determined as well. Correlation analysis and regression analysis were performed to analyze the relationship between PUFT and HDL level and cholesterol efflux capacity in all patients and subgroups.

**Results:**

Patients with higher PUFT have lower serum HDL level but increased cholesterol efflux capacity. Further analysis showed that PUFT negatively correlated with the serum HDL level in all patients, with no difference in groups divided by body mass index (BMI). In addition, PUFT was positively correlated with cholesterol efflux capacity in all patients. Multiple stepwise regression analysis showed an independent association of PUFT and serum HDL level and cholesterol efflux capacity.

**Conclusions:**

PUFT is closely correlated with the serum HDL level and cholesterol efflux capacity in patients with T2DM.

## 1. Background

Adipose tissue is commonly divided into visceral fat tissue and subcutaneous fat tissue based on anatomy and physiological features [1]. It has been reported that visceral fat was more related with metabolic and cardiovascular diseases [2, 3]. One research has demonstrated that adipose tissue in various locations had different enzyme histochemical traits [4], and further studies showed that tissue-specific adipocytes were likely to be associated with various diseases such as cardiovascular diseases, autoimmune diseases, infectious diseases, and cancers [5]. Para and perirenal fat is a fat pad surrounding the kidneys. It has a complete system of blood supply, lymph fluid drainage, and innervation [6]. Several studies have reported the close relationship between para and perirenal fat and cardiovascular diseases, including atherosclerosis, blood pressure, and endothelial damage [7–10].

High-density lipoprotein (HDL) is important in the metabolism of cholesterol [11]. Except that, accumulating evidence has showed that HDL was involved in the pathophysiology of diabetes, metabolic syndrome, and cardiovascular diseases [12]. Patients with type 2 diabetes mellitus (T2DM) are always accompanied by increased serum total cholesterol and decreased HDL level [13]. Our previous work has suggested that HDL dysfunction including cholesterol efflux capacity and inhibitory on endothelial lipase existed in patients with T2DM [14]. Some investigators have explored the possible mechanism of the reduction of HDL level and its function in T2DM [15, 16]. Our further research has showed that patients with T2DM have a lower serum HDL level and different components compared with non-T2DM patients [17]. In addition, adipokines including angiopoietin-like proteins have been thought to play an important role in the regulation of the HDL metabolism and its function [14, 17].

Several studies have explored the relationship between para and perirenal fat and serum HDL level in obese subjects, while the results were inconsistent. One study in obese patients found that perirenal fat thickness was lower and HDL cholesterol was higher after sleeve gastrectomy surgery [18]. While another study in obese animal found the contrary results [19]. Based on the above background, this study aimed to explore the relationship between para and perirenal fat and serum HDL level and cholesterol efflux capacity in patients with T2DM.

## 2. Methods and Materials

### 2.1. Study Subjects

In this cross-sectional study, we recruited a total of 58 Han Chinese subjects consecutively from the Beijing Luhe Hospital from September 2019 to January 2020. All subjects were diagnosed with T2DM according to the 1999 WHO criteria [20]. The exclusion criteria were as follows: (1) acute or chronic hepatitis, (2) severe abnormal liver function, defined as liver enzyme ≥3 times normal value, (3) usage of lipid-lower medicine, (4) severe abnormal renal function, defined as estimated glomerular filtration rate (eGFR) < 30 ml/min per 1.73 m^2^, and (5) in pregnancy or lactation.

### 2.2. Clinical and Laboratory Evaluation

Anthropometric and physical examinations were performed to collect bodyweight, height, and waist and hip circumference in all subjects. Glycated hemoglobin A1_C_ (HbA_1C_) (HA-8180, Japan), serum lipid profile, renal function, liver function, and albuminuria level (AU5811, USA) were measured in the clinical laboratory of our hospital. The area of VAT at the level of the umbilicus was measured via an abdominal dual BIA machine with DUALSCAN HDS-2000 (Omron Healthcare Co., Kyoto, Japan). eGFR were calculated using Chinese population-specific formula derived from the Modification of Diet in Renal Disease (MDRD) equation (GFR (mL/min/1.73 m^2^) = 175 × (Scr/88.4) − 1.154 × (age) − 0.203 × (0.742 if female)) [21].

### 2.3. Ultrasonographic Evaluation

Ultrasound examinations were performed by a single well-trained operator through a duplex Doppler (Hitachi Preirus, Hitachi, Japan). The operator was unaware of clinical data of all subjects. Para and perirenal fat thickness was measured via a 1–5 MHz transducer on the abdomen in the subjects with inspiration state in supine position. The probe was slowly moved laterally until the optimal position, at which the surface of the kidney was almost parallel to the skin. The pressure exerted on the probe was as minimal as possible, so that the fat layers were not compressed. Then, the para and perirenal fat thickness was measured from the inner side of the abdominal musculature to the surface of the kidney. The average of measurements on both sides was defined as the para-perirenal ultrasonographic fat thickness (PUFT).

### 2.4. Cholesterol Efflux Rate Examination

Cholesterol efflux assay was performed following the manual instruction (Biovision, CA, USA). RAW264.7 macrophages were plated at the density of 1 × 10^5^ cells/well in a 96-well white plate and maintained in DMEM plus 10% FBS (Sigma-Aldrich) for 2 h. The adherent cells were incubated with labeled cholesterol for 16 h and then exposed to 100 *μ*g/mL HDL for 4 h. The supernatant was transferred to a 96-well plate to measure the fluorescence (Ex/Em = 482/515 nm). The adherent cells were solubilized by cell lysis buffer to measure the fluorescence (Ex/Em = 482/515 nm). Cholesterol efflux % = fluorescence intensity of the media/(fluorescence intensity of the cell lysate + media) × 100%.

### 2.5. Statistical Analysis

Statistical analysis was first performed in the whole population, and then, in two groups divided based on PUFT (PUFT <2.08 cm, *n* = 29; PUFT ≥2.08 cm, *n* = 29, respectively). Continuous variables were expressed as mean ± standard deviation (SD), and the significance was tested by the *t*-test. Triglycerides and uric acid were expressed as median and interquartile range because of its skewed distribution, and significance was tested by the Mann–Whitney *U* test. The chi-square test was used to analyze the differences between male and female. The correlation between PUFT and anthropometric parameters with HDL level and cholesterol efflux rate was expressed by Pearson correlation coefficients (*r*). In addition, subgroup analysis was performed according to gender and BMI. Multiple linear regression analysis was used to evaluate the multivariate relationships between PUFT and HDL and cholesterol efflux. The stepwise multiple regression models were built on the whole population considering HDL and cholesterol efflux rate as response variables, respectively, and including PUFT, body mass index (BMI), and waist-to-hip ratio (WHR), VAT as explanatory variables. Collinearity was assessed by calculating the variance inflation factor (VIF). Variables with VIF ≥2 were excluded from the models. *P* < 0.05 was considered statistically significant in all statistical analyses. The statistical analyses were performed using SPSS statistics software package, version 20 (USA).

## 3. Results

### 3.1. Clinical Characteristics of Subjects

The clinical characteristics of the whole population are given in Table 1. All subjects were divided into two groups based on PUFT (<2.08 cm, *n* = 29, PUFT ≥2.08 cm, *n* = 29), and then, we compared the clinical characteristics of the population. HbA1c, triglycerides, uric acid, low-density lipoprotein (LDL), and eGFR showed no difference between the two groups. Subjects in the higher PUFT group had higher BMI, WHR, VAT, and longer diabetes course. The higher PUFT group has more smokers than the other group (14/29 vs. 7/29). Females had higher PUFT than that in males. Moreover, subjects who have higher PUFT had lower HDL cholesterol level (1.13 ± 0.26 vs. 0.94 ± 0.19, *p* < 0.01) and total lower cholesterol (40.76 ± 7.64 vs. 47.17 ± 9.49, *p* < 0.01) (Table 1).

### 3.2. Correlations between Serum HDL and Cholesterol Efflux Rate and Anthropometric and Metabolic Parameters

The univariate correlations of HDL and cholesterol efflux rate and anthropometric and metabolic parameters in the entire population are given in Table 2. Age and course of diabetes mellitus did not correlate with the serum HDL level and cholesterol efflux rate. BMI, WHR, and VAT were significantly negatively related with the serum HDL level, while less than PUFT (*r* = −0.42, *p* < 0.01). BMI was not significantly associated with cholesterol efflux rate, while WHR and VAT were positively related with cholesterol efflux rate, even less than PUFT (*r* = 0.43, *p* < 0.01).

**Figure 1:**
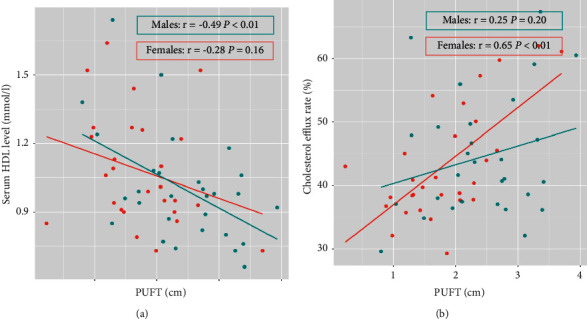
Correlations between para-perirenal ultrasonographic fat thickness (PUFT) and serum high-density lipoprotein (HDL) level (a) and cholesterol efflux capacity (b) in the subgroup divided by gender.

Then, we separately analyzed the relationship between serum HDL and cholesterol efflux rate and anthropometric and metabolic parameters in males and females (Supplementary Table 1). In males, PUFT were negatively related with the serum HDL level (*r* = −0.49, *p* < 0.01) but not cholesterol efflux rate (Figure 1). In females, BMI was negatively related with the serum HDL level (*r* = −0.40, *p* < 0.05), which is more related than PUFT (*r* = −0.28, *p* = 0.16). However, PUFT were positively correlated with cholesterol efflux rate (*r* = 0.65, *p* < 0.01) (Figure 1(b)), which is more obvious than BMI, WHR, and VAT.

**Figure 2:**
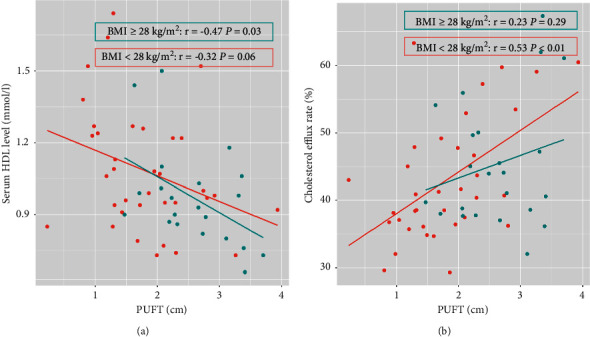
Correlations between para-perirenal ultrasonographic fat thickness (PUFT) and serum high-density lipoprotein (HDL) level (a) and cholesterol efflux capacity (b) in the subgroup divided by body mass index (BMI).

In addition, we performed a subgroup analysis of BMI (Supplementary Table 2). WHR was positively related with cholesterol efflux rate in patients with lower BMI (*r* = 0.39, *p* = 0.02), which is less than PUFT (*r* = 0.53, *p* < 0.01). While, no obvious relationship was seen in the patients with higher BMI (Figure 2).

### 3.3. Multivariate Model for HDL and Cholesterol Efflux Rate

Finally, we built the multiple linear regression model considering serum HDL level as response variable and including PUFT, BMI, WHR, visceral fat, and smoking status as explanatory variables. Only PUFT were independently associated with serum HDL level (*β* = −0.115, *p* = 0.009). Similarly, another multiple linear model was built considering cholesterol efflux rate as response variable, and only PULT was left in stepwise procedure (*β* = −4.027, *p* = 0.013). For the two models, collinearity was assessed by calculating VIF of the models, and there was no VIF >10 (Table 3).

## 4. Discussion

In our study, we found the independently and negatively association between PUFT and serum HDL level, and subgroup analysis showed the relation was more significant in male and individuals with obesity and diabetes. HDL cholesterol level and cholesterol efflux function of HDL play important roles in diabetes, metabolic syndrome, and cardiovascular disease [12]. Previous studies on different diet or materials regulating the lipid metabolism and para and perirenal fat tissue contents showed inconsistent results. Wang and colleagues found that a lactic acid bacterium (*Lactobacillus fermentum* CQPC05, LF-CQPC05) isolated from Sichuan pickles could decrease the values of hepatosomatic, epididymal fat, and perirenal fat indices and increase the HDL-C level in obese mice fed with a high-fat diet [22]. Moreover, lower perirenal fat thickness and higher HDL cholesterol were showed in morbidly obese patients after sleeve gastrectomy treatment in another study [18]. These studies showed that there may be a certain relationship existing between HDL cholesterol and perirenal fat. Manno and colleagues reported that para and perirenal fat but not epicardial adipose tissue was independently associated with insulin resistance and lower HDL cholesterol in a cohort of 102 uncomplicated overweight and obese patients [23].

HDL dysfunction in patients with T2DM has been revealed in our previous work [14]. One study measured serum endocan, lipid parameters, and lipoprotein subclasses in patients with T2DM and diabetes-free participants found that lower HDL3b proportions were associated with higher endocan levels in population with T2DM [24]. Multiple studies indicated that higher level of HDL cholesterol did not lead to expected clinical benefits of the cardiovascular system, which may due to the HDL dysfunction or altered HDL function [25]. HDL function includes cholesterol efflux capacity, anti-inflammatory, antithrombotic, antioxidant, and vascular protective effects [26]. The cholesterol efflux capacity of HDL has been considered as a cardiovascular disease risk predictor independently of HDL cholesterol [27]. We evaluated cholesterol efflux capacity of HDL by in vitro assay which measured the ability of an individual's HDL to promote cholesterol efflux from cholesterol donor cells such as macrophages. In this study, we found that PUFT was positively correlated with cholesterol efflux capacity of HDL in 58 subjects. PUFT was positively correlated with cholesterol efflux capacity of HDL in subjects with BMI <28 kg/m^2^, but has no correlation in subjects with BMI ≥28 kg/m^2^. Moreover, this positive relationship can also be seen in the female subgroup but not in males. The reason may be that the increased PUFT could stimulate the cholesterol efflux capacity of HDL in the condition of normal perirenal fat distribution. However, when perirenal fat accumulated abnormally, the cholesterol efflux capacity of HDL decreased due to the change of HDL components such as reduced level of apoAI, apoAII, and higher levels of serum amyloid A (SAA). The apoAI is a main component in cholesterol efflux capacity of HDL. SAA is an inhibitor of HDL anti-inflammatory function. It was reported that HDL function was decreased in CKD patients. HDL components isolated from CKD patients were changed by biochemical and mass spectrometry analyses, which showed reduced apoAI, apoAII, apoM, and paraoxonase level and increased serum amyloid A (SAA), apoCII, and lipoprotein-associated phospholipase A2 level [28]. Our previous work indicated that the decrease of angiopoietin-like protein 3 level might contribute to the reduced capacity of cholesterol efflux in female patients with T2DM [17]. It has been confirmed that angiopoietin-like protein 3 is derived from liver and fat tissue; therefore, we guess that para and perirenal fat tissue distribution may influence the HDL and its function via adipokines.

There are some limitations in the study. First, the study is a cross-sectional study, and it could not establish the causal relation between PUFT and HDL levels and cholesterol efflux capacity. A follow-up visit cohort and basic experiment are needed to verify the results. Second, we measured PUFT by ultrasonography instead of computed tomography (CT). Since, ultrasound is more convenient and simpler than CT in clinical practice. To control variation, we have one fixed and well-trained operator measuring the PUFT.

## 5. Conclusions

In summary, our study showed that PUFT was independently and negatively associated with the serum HDL level. PUFT was independently and positively related with cholesterol efflux capacity. However, the underlying mechanism still need further research to illuminate it.

## Figures and Tables

**Table 1:** The basic clinical characteristics of the population.

	Whole population (*n* = 58)	PUFT <2.08 cm (*n* = 29)	PUFT ≥2.08 cm (*n* = 29)
Gender (M/F)	29/29	10/19	19/10^*∗*^
Age (years)	54.69 ± 14.50	58.69 ± 12.29	50.69 ± 15.62^*∗*^
Course of diabetes (years)	7.47 ± 6.64	9.38 ± 6.73	5.55 ± 6.08^*∗*^
Smoker	21/58	7/29	14/29^*∗*^
BMI (kg/m^2^)	26.91 ± 4.75	24.65 ± 3.23	29.16 ± 5.00^*∗∗*^
WHR	0.97 ± 0.56	0.95 ± 0.41	0.98 ± 0.58^*∗*^
VAT (cm^2^)	108.32 ± 40.31	90.31 ± 33.93	129.47 ± 37.33^*∗∗*^
PUFT (cm)	2.12 ± 0.82	1.46 ± 0.45	2.79 ± 0.51^*∗∗*^
HbA1c (%)	9.62 ± 2.13	9.52 ± 2.28	9.73 ± 2.01
Total cholesterol (mmo/l)	4.28 ± 1.32	4.65 ± 1.61.64	3.87 ± 0.66^*∗*^
LDL cholesterol (mmol/l)	2.78 ± 1.02	3.03 ± 1.25	2.52 ± 0.62
HDL cholesterol (mmol/l)	1.04 ± 0.24	1.13 ± 0.26	0.94 ± 0.19^*∗∗*^
Cholesterol efflux rate (%)	43.92 ± 9.17	40.76 ± 7.64	47.17 ± 9.49^*∗∗*^
Triglycerides (mmol/l)	1.56 (1.09, 2.12)	1.29 (0.99, 2.06)	1.77 (1.27, 2.17)
Uric acid (umol/l)	333.00 (251.50, 399.75)	306.83 ± 83.81	358.22 ± 112.31
eGFR (ml/min per 1.73 m^2^)	97.92 ± 29.88	98.53 ± 33.96	97.31 ± 25.75

BMI, body mass index; WHR, waist-to-hip ratio; VAT, visceral fat tissue; PUFT, para-perirenal ultrasonographic fat thickness; HbA1c, glycated hemoglobin A1_C_; LDL, low-density lipoprotein; HDL, high-density lipoprotein; eGFR, estimated glomerular filtration rate. ^*∗*^*P* < 0.05. ^*∗∗*^*P* < 0.01.

**Table 2:** Correlations between para-perirenal ultrasonographic fat thickness and anthropometric parameters with HDL level and cholesterol efflux rate in the whole population.

	HDL level (mmol/l)		Cholesterol efflux rate (%)	
	*r*	*P*	*r*	*P*
Age (years)	0.20	0.14	−0.12	0.39
Course of diabetes (years)	0.07	0.61	−0.25	0.06
BMI (kg/m^2^)	−0.34	0.01	0.23	0.09
WHR	−0.14	0.31	0.34	<0.01
VAT (cm^2^)	−0.24	0.10	0.31	0.03
PUFT (cm)	−0.42	<0.01	0.43	<0.01

BMI, body mass index; WHR, waist-to-hip ratio; VAT, visceral fat tissue; PUFT, para-perirenal ultrasonographic fat thickness; HDL, high-density lipoprotein.

**Table 3:** Independent multiple linear regression analysis of serum HDL level and cholesterol efflux rate. The other variables included in the models are described in the text.

	*β*	*P*
HDL (mmol/l)^a^		
Model (*R*^2^ = 0.176)		
PUFT (cm)	−0.115	0.009

Cholesterol efflux rate (%)^b^		
Model (*R*^2^ = 0.121)		
PUFT (cm)	4.027	0.013

HDL, high-density lipoprotein; PUFT, para-perirenal ultrasonographic fat thickness.

## Data Availability

The datasets used and/or analyzed during the current study are available from the corresponding author upon request.

## List of abbreviations

T2DM: Type 2 diabetes mellitus
BMI: Body mass index
WHR: Waist-to-hip ratio
VAT: Visceral fat tissue
PUFT: Para-perirenal ultrasonographic fat thickness
HbA1c: Glycated hemoglobin A1_C_
LDL: Low-density lipoprotein
HDL: High-density lipoprotein
eGFR: Estimated glomerular filtration rate.

## Supplementary Materials

Supplementary Materials Supplementary Table 1. Correlations between anthropometric parameters and HDL level and cholesterol efflux rate in the gender subgroup. Supplementary Table 2. Correlations between anthropometric parameters and HDL level and cholesterol efflux rate in the BMI subgroup. .
