# Supplementary material for: Relationship of Para and Perirenal Fat and High-Density Lipoprotein and Its Function in Patients with Type 2 Diabetes Mellitus
Source: Int J Endocrinol. 2021 Dec 22;2021:9286492. doi: 10.1155/2021/9286492 (PMC8716211; doi:10.1155/2021/9286492)
Supplement: Supplementary Materials — Supplementary Table 1. Correlations between anthropometric parameters and HDL level and cholesterol efflux rate in the gender subgroup. Supplementary Table 2. Correlations between anthropometric parameters and HDL level and cholesterol efflux rate in the BMI subgroup. . [file 9286492.f1.docx]

**Supplementary tables**

Supplementary table 1 Gender subgroup correlation analysis

|  | HDL level (mmol/L) | | Cholesterol efflux rate (%) | |
| --- | --- | --- | --- | --- |
|  | r | *p* | *r* | *p* |
| **Male** |  |  |  |  |
| BMI (kg/m^2^) | -0.24 | 0.21 | 0.06 | 0.74 |
| WHR | -0.18 | 0.36 | 0.30 | 0.11 |
| VAT (cm^2^) | -0.20 | 0.36 | 0.12 | 0.59 |
| PUFT (cm) | -0.49 | <0.01 | 0.25 | 0.20 |
| **Female** |  |  |  |  |
| BMI (kg/m^2^) | -0.40 | 0.04 | 0.41 | 0.03 |
| WHR | 0.03 | 0.88 | 0.41 | 0.03 |
| VAT (cm^2^) | -0.26 | 0.22 | 0.61 | <0.01 |
| PUFT (cm) | -0.28 | 0.16 | 0.65 | <0.01 |

BMI, body mass index; WHR, waist-to-hip ratio; VAT, visceral fat tissue; PUFT, para-perirenal ultrasonographic fat thickness; HDL, high density lipoprotein.

Supplementary table 2 Subgroup correlation analysis of body mass index (BMI)

|  | HDL level (mmol/L) | | Cholesterol efflux rate (%) | |
| --- | --- | --- | --- | --- |
|  | r | *p* | *r* | *p* |
| **BMI<28** |  |  |  |  |
| BMI (kg/m^2^) | -0.33 | 0.05 | 0.22 | 0.20 |
| WHR | 0.02 | 0.91 | 0.39 | 0.02 |
| VAT (cm^2^) | -0.21 | 0.27 | 0.33 | 0.07 |
| PUFT (cm) | -0.32 | 0.06 | 0.53 | <0.01 |
| **BMI>=28** |  |  |  |  |
| BMI (kg/m^2^) | -0.21 | 0.37 | 0.19 | 0.39 |
| WHR | -0.14 | 0.55 | 0.24 | 0.27 |
| VAT (cm^2^) | 0.06 | 0.81 | 0.13 | 0.59 |
| PUFT (cm) | -0.47 | 0.03^*^ | 0.23 | 0.29 |

BMI, body mass index; WHR, waist-to-hip ratio; VAT, visceral fat tissue; PUFT, para-perirenal ultrasonographic fat thickness; HDL, high density lipoprotein.
